# Draft Genome Sequence of *Staphylococcus aureus* subsp. *aureus* Strain HG003, an NCTC8325 Derivative

**DOI:** 10.1128/genomeA.00855-14

**Published:** 2014-08-28

**Authors:** Mohamed Sassi, Brice Felden, Yoann Augagneur

**Affiliations:** Rennes University, Inserm U835 Biochimie Pharmaceutique, Rennes, France

## Abstract

We report the draft genome sequence of a *Staphylococcus aureus* NCTC8325 derivative, strain HG003. HG003 contains functional global regulators *rsbU* and *tcaR* and is therefore considered as a reference for studies of regulation and virulence. The genome is composed of 2,797,898 bp and will be essential for subsequent RNAseq analysis.

## GENOME ANNOUNCEMENT

*Staphylococcus aureus* is a serious opportunistic human pathogen responsible for food poisoning, skin infections, and multi-resistant nosocomial infections (1). Strain HG003 is considered as a reference model by the scientific community, with functional *rsbU* and *tcaR* global regulators (2). However, while its parental strain NCTC8325 was isolated in 1960 from a sepsis patient (3) and its genome sequenced in 2001 (4), strain HG003 was generated with molecular tools only in 2010 (2). Staphylococci have high genomic plasticity (i.e., by recombination and by elevated mutation frequency) (5–8). Therefore, there is a need to obtain an accurate genome sequence of this reference strain, especially for those working on *S. aureus* physiology and virulence, and also because there is growing interest in high-throughput RNA sequencing. Here, we present the draft genome of reference strain HG003.

Genomic DNA was isolated from strain HG003 grown on BHI agar plate at 37°C using the Wizard genomic DNA purification kit (Promega) following the manufacturer recommendations for an efficient lysis of *S. aureus*. Subsequently, genomic DNA was precipitate with sodium acetate and washed two times with ethanol 70% (vol/vol). DNA was sheared using a Covaris M220 to obtain a peak at 600 pb. Unwanted small and large fragments were removed by size-selection using AMPure XP beads (Beckman Coulter). DNA library was prepared using the NEBNext ultra DNA library prep kit for Illumina (NEB) and sequenced as paired-end reads using an Illumina MiSeq platform and a MiSeq reagent kit v3 (600 cycles) (Illumina, Inc., San Diego, CA). The run generated 1,826,904 reads (548 Mb of sequence data) with 95× coverage.

Illumina reads, trimmed using Trimmomatic (9) and quality filtered using Fastx-toolkit (http://hannonlab.cshl.edu/fastx_toolkit/), were assembled using the Spades software (10, 11).

These analyses yielded 19 scaffolds of 20 contigs containing 2,797,898 bp, for a 32.74% G+C content, representing 99.14% of the completed genome using its parental strain *S. aureus* subsp. *aureus* NCTC8325 as a reference. We first verified that *rsbU* and *tcaR* genes were actually restored in the clone sequenced. However, the average nucleotide identity between the two strains is of 99.97% suggesting that other mutations are present in strain HG003 compared to NCTC8325.

The genome sequence was annotated by the NCBI Prokaryotic Genomes Annotation Pipeline (PGAP) (12). The genome comprises 2,647 genes, 11 predicted RNAs (5S, 16S, and 23S), 50 tRNAs, one transfer-messenger RNA (tmRNA). Among the 2,647 genes, 2,573 (97.2%) were found to encode putative proteins and 488 (18.96%) were assigned as hypothetical proteins. The genome encodes three intact *Staphylococcus* complete phages and four questionable clustered regularly interspaced short palindromic repeats (CRISPRs), according to the PHAge Search Tool (Phast) (13) and the CRISPERfinder online software (14).

### Nucleotide sequence accession numbers.

This whole-genome shotgun project has been deposited at DDBJ/EMBL/GenBank under the accession no. JPPU00000000. The version described in this paper is version JPPU01000000.
